# Photocurrent enhancement mechanisms in bilayer nanofilm-based ultraviolet photodetectors made from ZnO and ZnS spherical nanoshells

**DOI:** 10.1186/1556-276X-9-388

**Published:** 2014-08-11

**Authors:** Lin Peng, Sancan Han, Xinhua Hu

**Affiliations:** 1 Department of Materials Science, Fudan University, Shanghai 200433, People’s Republic of China; 2 Department of Physics, Shanghai University of Electric Power, Shanghai 200090, People’s Republic of China

**Keywords:** Hollow-sphere bilayer nanofilm, Ultraviolet light photodetector, Whispering gallery mode resonances

## Abstract

Hollow-sphere bilayer nanofilm-based ultraviolet light photodetectors made from ZnO and ZnS spherical nanoshells show enhanced photocurrent, which are comparable to or even better than those of other semiconductor nanostructures with different shapes. In this work, the photocurrent enhancement mechanisms of these bilayer nanofilm-based ultraviolet light photodetectors are explained, which could be attributed to the strong light absorption based on the whispering gallery mode resonances, the separation of the photogenerated carriers through the internal electric field within the bilayer nanofilms, the hopping-like electrical transport, and the effective charge injection from Cr/Au contacts to the nanofilms.

## Background

Monodisperse spherical nanoshells (or called hollow spheres) have attracted considerable interest due to their well-defined morphology, uniform size, low density, high surface area, and potential applications such as protection of biologically active agents, waste removal, and so on [1-3]. On the other hand, some novel nanodevices with high performance have been constructed using semiconducting hollow spheres as the building blocks [4,5]. For instance, dye-sensitized solar cells using electrodes consisting of nanoembossed TiO_2_ hollow spheres exhibit outstanding light-harvesting efficiency [4]. Nanocrystalline silicon (nc-Si) solar cells based on the hollow-sphere nc-Si nanofilm are constructed, which exploit the low-quality-factor whispering gallery modes (WGMs) in hollow spheres to dramatically enhance broadband absorption [5]. Most of the incoming light couples into the WGMs in the hollow spheres and circulates in the active material with a considerably longer path length than that of the same material in the form of a planar film. Such light-trapping structure is an essential design consideration for high-performance photodetectors (PDs), as well as other optical devices such as solar cells.

Recently, we have developed a self-assembly strategy at the immiscible oil-water interface to fabricate monolayer hollow-sphere nanofilm-based devices, such as ultraviolet (UV) light PDs and electrical resistive switching memory devices [6-9]. On the other hand, we also use the self-assembly strategy to construct hollow-sphere bilayer nanofilm-based UV PD devices, which show improved optoelectronic properties [10]. Hollow-sphere bilayer nanofilm-based UV PDs using abundant wurtzite ZnO and ZnS hollow nanospheres as the building blocks were constructed by the oil-water interfacial self-assembly strategy. These hollow-sphere nanofilm-based UV PDs showed high sensitivity, good stability, and fast response times, which are comparable to or even better than those of other ZnO nanostructures with different shapes [10-17]. It is quite promising for applications such as optical communications, flame sensing, missile launch, and so forth. However, we need to explore the photocurrent enhancement mechanisms in the bilayer nanofilm-based UV PDs made from ZnO and ZnS hollow spheres in order to further improve their optoelectronic properties. We believe that the photogenerated charges are extracted from these devices to not simply produce the photocurrent but instead cause some new changes in these devices which impel further free carriers to be generated and transported through the devices. In this work, the photocurrent enhancement mechanisms of these bilayer nanofilm-based UV PDs are explained. Especially, we prove a concept for light trapping in the hollow-sphere nanofilm-based UV PDs through the use of wavelength-scale resonant hollow spheres that support WGMs to enhance absorption and photocurrent. We numerically demonstrate this enhancement using full-field finite element method (FEM) simulations of hollow-sphere nanofilm-based UV PDs. It is proved that the WGM is an important concept for the manufacturing of the hollow-sphere nanofilm-based UV PDs, which facilitates the coupling of light into the resonant modes and substantial enhancement of the light path in the active materials, thus dramatically enhancing absorption and photocurrent.

## Methods

**Figure 1:**
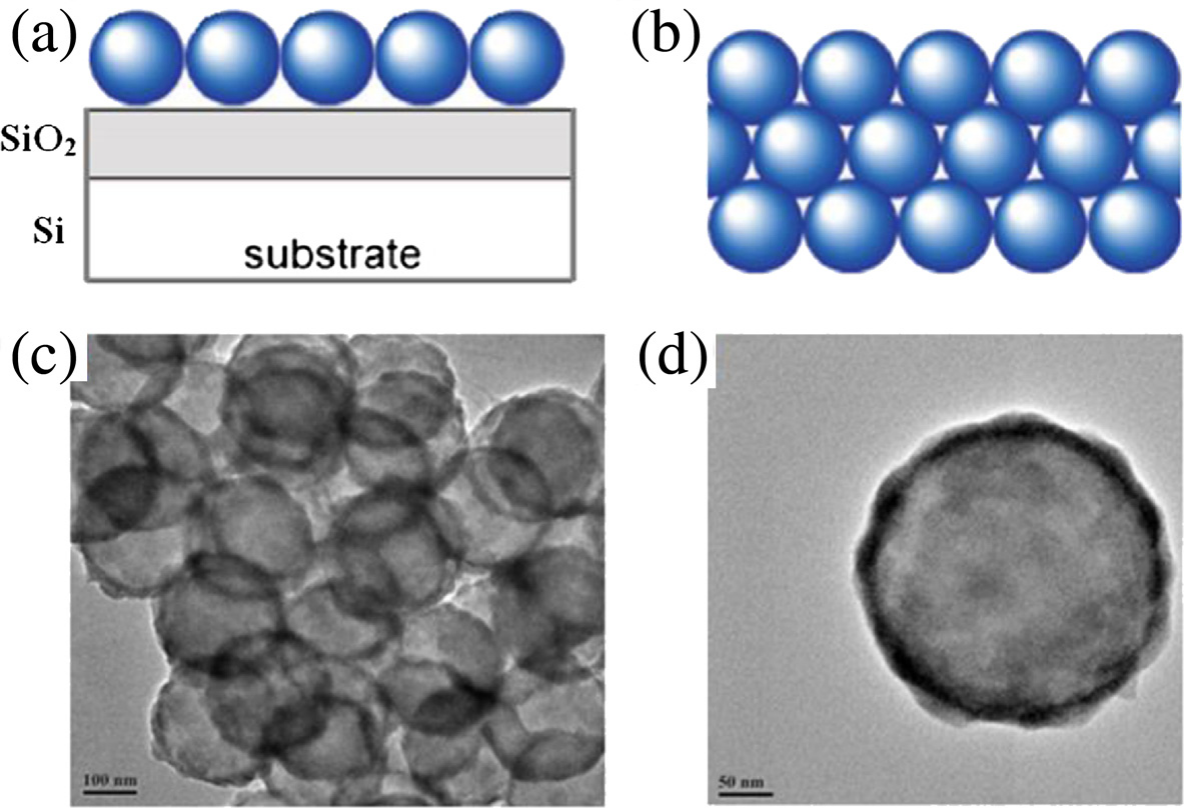
**Images of the ZnO hollow-sphere nanofilm and typical TEM image of a ZnO hollow sphere. (a)** Side view of the ZnO hollow-sphere nanofilm deposited on Si (100)/SiO_2_. **(b)** Front view of the ZnO hollow-sphere nanofilm deposited on Si (100)/SiO_2_. **(c)** Typical TEM image of the ZnO hollow-sphere nanofilm. **(d)** Typical TEM image of a ZnO hollow sphere.

The preparation of hollow spheres is quite simple and scalable without the need for lithography. Figure 1a depicts a ZnO hollow-sphere nanofilm-based UV PD. Well-defined polystyrene (PS)/ZnO core/shell nanospheres were prepared and then self-assembled at a hexane-water interface to form a precursor film. The precursor core/shell film was then transferred onto a Si substrate covered with a 200-nm-thick layer of SiO_2_. Annealing this precursor film under optimal conditions, a ZnO hollow-sphere nanofilm with a densely packed network structure was obtained. The front view is depicted in Figure 1b. Finally, after a pair of Cr/Au electrodes was deposited on the as-transformed ZnO hollow-sphere nanofilm on a SiO_2_/Si substrate using an Au microwire as the mask, a UV PD was successfully constructed [8,10]. Figure 1c,d shows the typical transmission electron microscopy (TEM) images of the ZnO hollow spheres. One can see that the thickness of the ZnO shell is about 20 nm (average outer radius *R*_out_ = 120 nm and inner radius *R*_in_ = 100 nm). On the other hand, well-ordered ZnO/ZnS bilayer films were also fabricated by oil-water interfacial self-assembly. First, a large number of PS/ZnO core-shell microspheres were self-assembled at a hexane-water interface. Second, another monolayer film, using PS/ZnS core-shell microspheres, was fabricated at the hexane-water interface in another vessel. This monolayer was then transferred onto the substrate covered with the first PS/ZnO monolayer. The stacking sequence of these bilayer nanofilms can be easily tailored through the layer-by-layer deposition order. Then, we prepared two bilayer nanofilms composed of hollow microspheres with different stacking sequences. These two bilayer nanofilms are here referred to as ‘ZnO/ZnS/SiO_2_/Si (ZnO/ZnS)’ and ‘ZnS/ZnO/SiO_2_/Si (ZnS/ZnO).’ For the optoelectronic property measurements, a drastic increase of current up to 2.94 μA is detected in the ZnO/ZnS device at an applied voltage of 5.0 V when the wavelength of light source is 370 nm, while the current for the ZnS/ZnO device increases drastically to 18 μA under the same conditions [10]. At the same time, we note that the current of the ZnO/ZnS device is about one sixth of that of the ZnS/ZnO device, although it is higher than that of monolayer-based PDs [8].

## Results and discussion

The optical and electrical measurements provide insight into the photoconductive mechanism in ZnO/ZnS (or ZnS/ZnO) bilayer nanofilm devices, including the light absorption, the generation of free carriers, the charge transport, and the charge injection from metal contacts to the nanofilms. We note a remarkable enhancement in photocurrent for the bilayer nanofilm-based UV PDs, so we require a mechanism where the photogenerated charges are extracted from the devices not simply to produce the photocurrent but instead cause some new changes in these devices which impel further free carriers to be generated and transported through the devices.

**Figure 2:**
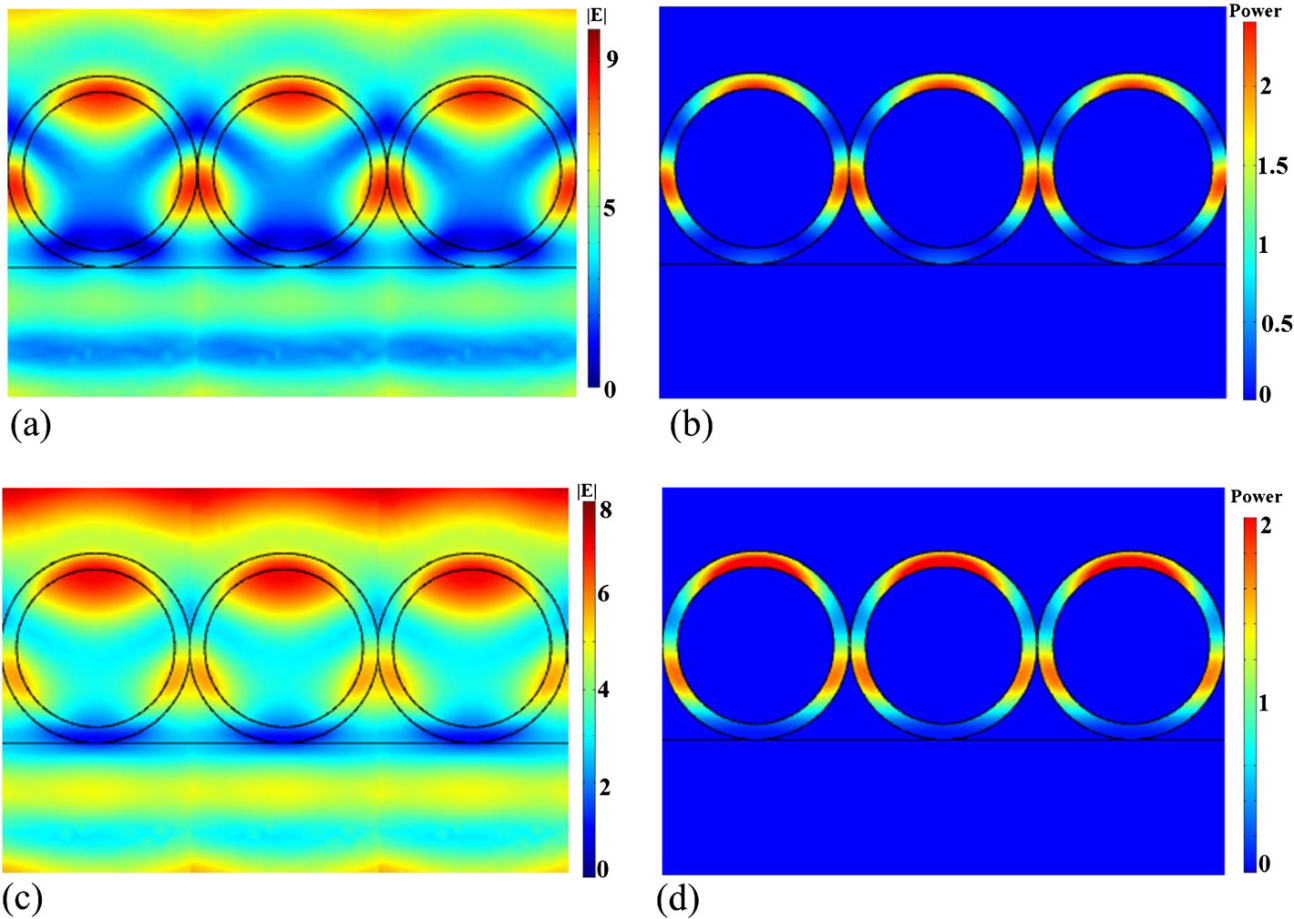
**Electric field (| *E *|) distribution and absorption power distribution. (a)** Electric field (|*E*|) distribution based on full-wave simulation of electromagnetic waves coupled with the ZnO hollow-sphere nanofilm at 370 nm. **(b)** Power distribution of the ZnO hollow-sphere nanofilm at 370 nm. **(c)** Electric field (|*E*|) distribution based on full-wave simulation of electromagnetic waves coupled with the ZnO hollow-sphere nanofilm at 350 nm. **(d)** Power distribution of the ZnO hollow-sphere nanofilm at 350 nm.

**Figure 3:**
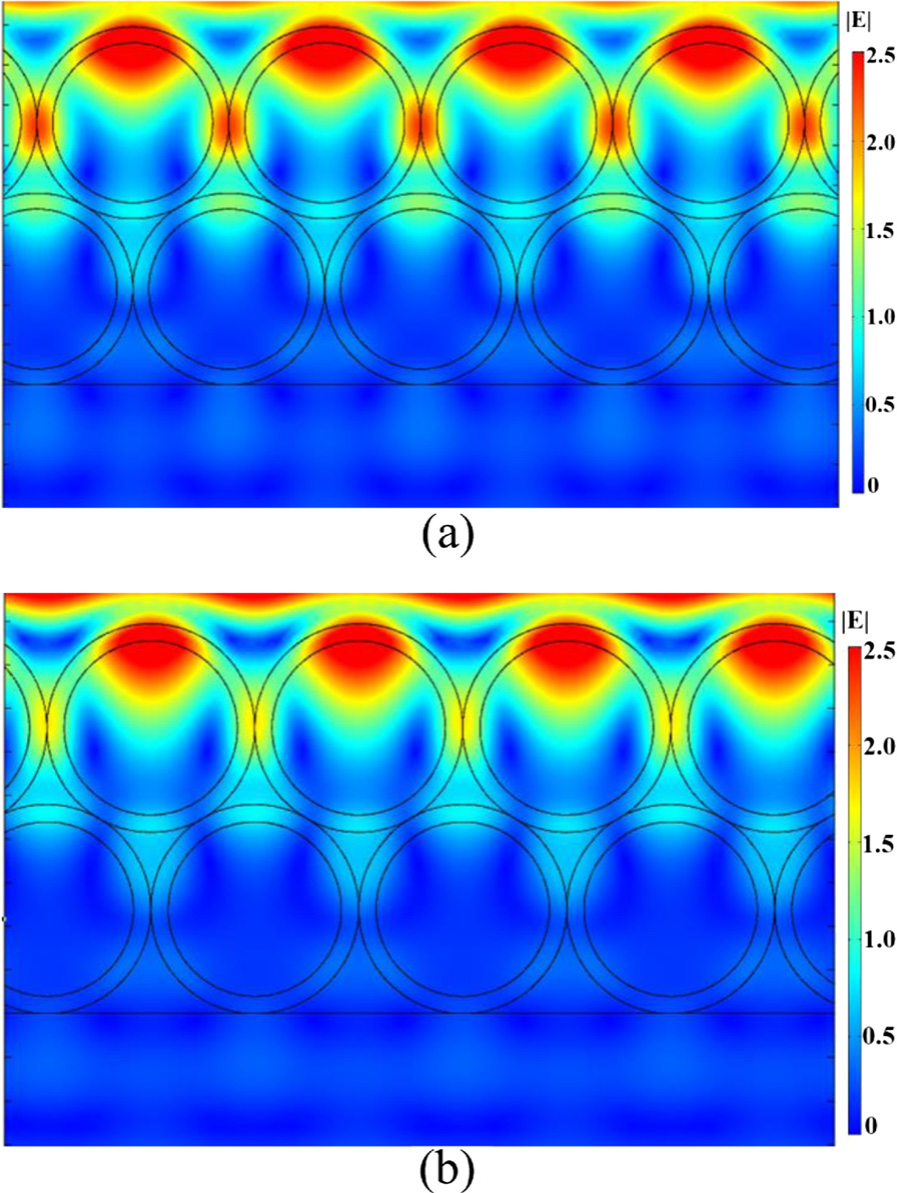
**Electric field (|*E*|) distribution. (a)** Electric field (|*E*|) distribution based on full-wave simulation of electromagnetic waves coupled with the ZnO/ZnS hollow-sphere nanofilm at 370 nm. **(b)** Electric field (|*E*|) distribution based on full-wave simulation of electromagnetic waves coupled with the ZnS/ZnO hollow-sphere nanofilm at 370 nm.

**Figure 4:**
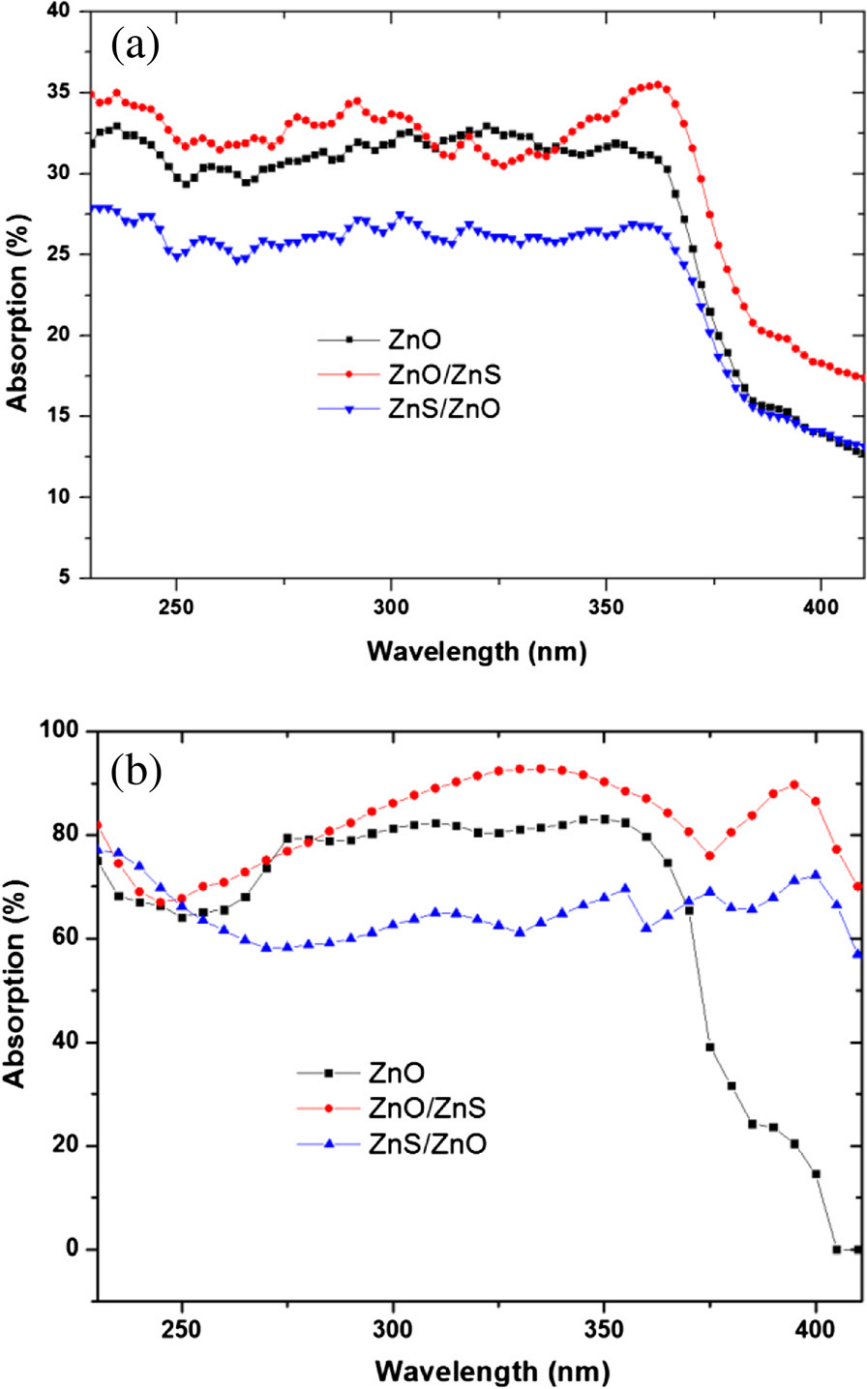
**UV-vis absorption spectra. (a)** UV-vis absorption spectra of the ZnO, ZnO/ZnS, and ZnS/ZnO nanofilms. **(b)** Absorption spectra simulated from the ZnO, ZnO/ZnS, and ZnS/ZnO nanofilm structures.

Light absorption based on the WGM resonances in the hollow-sphere nanofilm could be the most important factor. Light scattering by a dielectric concentric hollow sphere has been studied previously and can be formally solved [18,19]. To better understand the light-trapping effect, we performed 3D full-field FEM simulations for the hollow-sphere ZnO nanofilm structure to determine the expected light absorption based on the WGM resonances. The time average power loss was calculated using the equation *Q* = *cϵ*_0_*nα*|*E*|^2^/2, where *c* is the speed of light in free space; *ϵ*_0_ is the permittivity of free space; *α* is the absorption coefficient, with *n* being the real part of the complex refractive index; and *E* is the electric field. Figure 2 shows the amplitude of the WGM electric field pattern and the absorption power at 350 and 370 nm for the hollow-sphere ZnO nanofilm structure, respectively. Incident plane waves come from the top side with the electric field perpendicular to the paper plane and with an amplitude of 1 W. Figure 2 shows that most of the light is confined and guided along the shells instead of directly passing through the shells. The round shape of the shell forms a closed path for light and causes resonance at the given frequencies. The circulation of electromagnetic waves inside the nanoshell leads to the accumulation of electromagnetic energy inside the active material. Therefore, the resonant modes in the shells enhance light trapping and absorption and then photocurrent.For the ZnO/ZnS and ZnS/ZnO bilayer nanofilms, electrical field patterns in the cross section along one of the shell rows are given in Figure 3. The simulation result shows that light is mainly guided inside the shells of the top layer nanofilm, and strong light absorption based on the WGM resonances is observed. Furthermore, we measure the UV-visible (UV-vis) absorption spectra of the ZnO/ZnS, ZnS/ZnO, and ZnO nanofilms in Figure 4a. One can see that the absorbance is more prominent in the ZnO/ZnS bilayer nanofilm, but it is about one third of the simulated absorption spectrum of the ZnO/ZnS bilayer nanofilm (see Figure 4b). This could mainly be caused by the scattering due to the imperfect arrays (or defects) in our samples (see Figure 1c), which weaken the light absorption based on the WGM resonances to some extent. The big challenge is how to use this interfacial self-assembly strategy to grow high-quality multilayer nanofilms with uniform coverage ratios and smooth surfaces suitable for use in these optoelectronic devices. Even so, we could make a conclusion that the use of wavelength-scale resonant hollow spheres in our bilayer nanofilms supports whispering gallery modes to enhance light absorption and then photocurrent.

**Figure 5:**
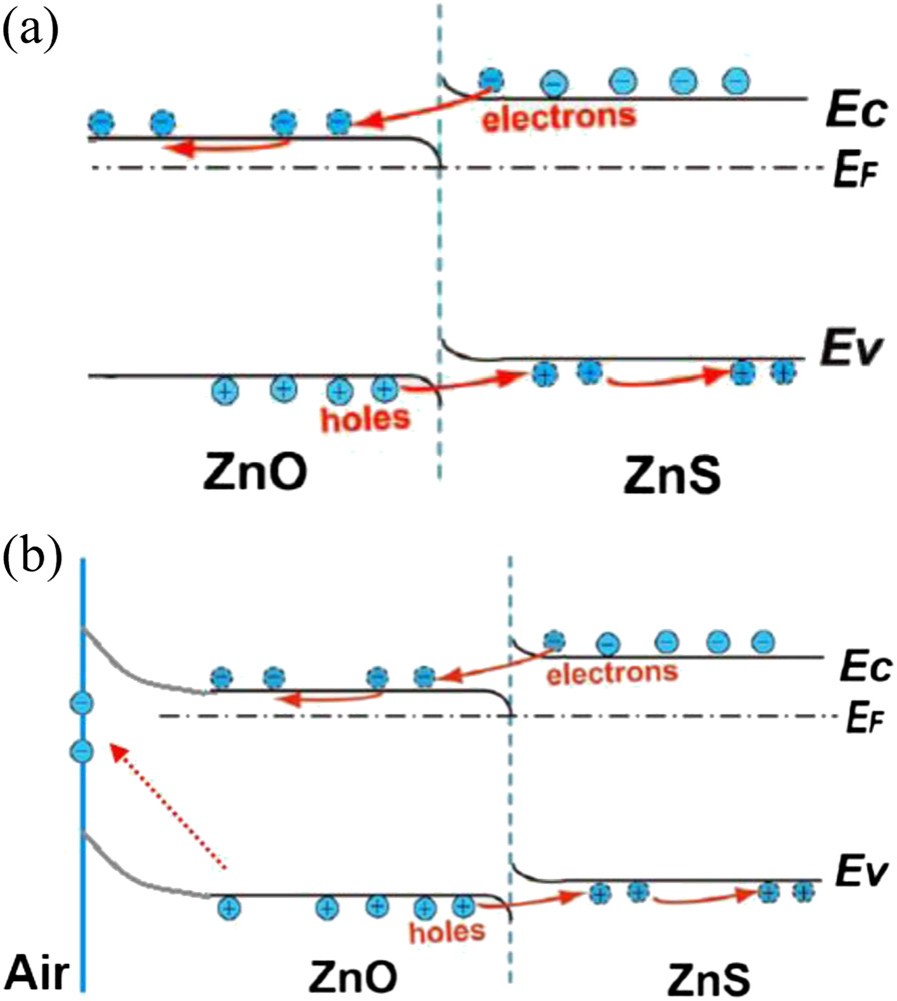
**Energy level diagrams and the charge transfer process under UV light illumination. (a)** ZnS/ZnO heterojunction. **(b)** ZnO/ZnS heterojunction.

It is very important to effectively separate the photogenerated carriers within the optoelectronic devices. The ZnO/ZnS and ZnS/ZnO bilayer nanofilms made of ZnO and ZnS hollow nanospheres can be regarded as heterostructured assemblies. The position of the valence band (VB) energy level of ZnS is about 0.6 eV higher than that of ZnO, and a type II heterostructure with a staggered alignment at the heterojunction is formed in our bilayer nanofilms [20]. The presence of an internal electric field due to the band bending at the heterostructure interface facilitates the separation of photogenerated carriers (see Figure 5). By the effective absorption of photons with energy greater than the bandgap, electron-hole pairs are photogenerated in semiconductor nanostructures. As the electron affinity of ZnO is higher than the affinity of ZnS [21], the photogenerated holes will migrate to the ZnS layer, while the free electrons will be transferred into the ZnO layer and then transported to the electrode. Therefore, we suggest that the increase of the photocurrent in the ZnS/ZnO device also strongly depends on the effective separation of the photogenerated carriers through the internal electric field in the bilayer nanofilm which significantly reduces the electron-hole recombination ratio (see Figure 5a), resulting in a much higher photocurrent compared with that of the monolayer-film device [8]. Compared with the ZnS/ZnO device, however, the ZnO/ZnS device exhibits a significant difference. As the top ZnO layer in the ZnO/ZnS device is exposed to the air, oxygen molecules are adsorbed onto the ZnO surface by capturing free electrons from the ZnO layer [O_2_(g) + e^−^ → O_2_^−^(ad)], which forms a low-conductivity depletion layer near the surface [13], creating the upward surface band bending (see Figure 5b). Under UV illumination, electron-hole pairs in the ZnO/ZnS heterostructure are photogenerated. Photoexcited holes move toward the surface along the potential gradient produced by band bending at the surface and discharge the negatively charged oxygen molecules adsorbed at the surface [h^+^ + O_2_^−^(ad) → O_2_(g)]. The chemisorption and photodesorption of oxygen molecules from the ZnO surface, to some extent, weaken the internal electric field which is built due to the band bending at the ZnO/ZnS heterostructure interface, thus impeding the separation of the photogenerated carriers within the ZnO/ZnS heterostructure and leading to the decreased photocurrent. In spite of this, the importance of the internal electric field on the separation of photogenerated carriers in the ZnO/ZnS heterostructure can still not be ignored, which still leads to the higher photocurrent compared with that of the monolayer-film device [8]. These predictions are in good agreement with our experimental results.

In addition, in the UV PDs based on the hollow-sphere bilayer nanofilms, the charge transfer between two neighboring hollow spheres is hopping-like due to the existence of physical boundaries [8]. In these devices where the current is space charge limited, it is easy to see that decreasing the trapping of free charges will lead to an increase in effective mobility and hence current. For the electrical transport through the interface between the Cr/Au electrode and the semiconductor, the formed ohmic or injection-type electric contacts in these UV PDs also contribute to the high photoresponsivity [8,10,22-24].

## Conclusions

In conclusion, we have demonstrated that the UV PDs can be conveniently fabricated using the hollow-sphere bilayer nanofilms. The UV PDs show high performance, which could be attributed to the strong light absorption based on the WGM resonances, the separation of the photogenerated carriers through the internal electric field within the bilayer nanofilms, the hopping-like electrical transport, and the effective charge injection from gold contacts to the nanofilms. It is quite promising for applications such as optical communications, flame sensing, and missile launch.

## Competing interests

The authors declare that they have no competing interests.

## Authors’ contributions

LP participated in the simulation studies and drafted the manuscript, SH participated in the design of the experiment, and XH participated in the revision of the manuscript. All authors read and approved the final manuscript.
